# Structures and Functions of Snake Venom Metalloproteinases (SVMP) from *Protobothrops* venom Collected in Japan

**DOI:** 10.3390/molecules22081305

**Published:** 2017-08-04

**Authors:** Etsuko Oyama, Hidenobu Takahashi

**Affiliations:** Department of Hygienic Chemistry, Meiji Pharmaceutical University, 2-522-1 Noshio, Kiyose-shi, Tokyo 204-8588, Japan; h-taka@my-pharm.ac.jp

**Keywords:** snake venom metalloproteinase, fibrinolytic/fibrinogenolytic activity, hemorrhagic activity, inhibitior of platelet aggregation

## Abstract

Snake venom metalloproteinases (SVMP) are widely distributed among the venoms of Crotalinae and Viperidae, and are organized into three classes (P-I, P-II and P-III) according to their size and domain structure. P-I SVMP are the smallest SVMP, as they only have a metalloproteinase (M) domain. P-II SVMP contain a disintegrin-like (D) domain, which is connected by a short spacer region to the carboxyl terminus of the M domain. P-III SVMP contain a cysteine-rich (C) domain, which is attached to the carboxyl terminus of the D domain. Some SVMP exhibit hemorrhagic activity, whereas others do not. In addition, SVMP display fibrinolytic/fibrinogenolytic (FL) activity, and the physiological functions of SVMP are controlled by their structures. Furthermore, these proteinases also demonstrate fibrinogenolytic and proteolytic activity against synthetic substrates for matrix metalloproteinases and ADAM (a disintegrin and metalloproteinase). This article describes the structures and FL, hemorrhagic, and platelet aggregation-inhibiting activity of SVMP derived from *Protobothrops* snake venom that was collected in Japan.

## 1. Introduction

Marked hemorrhaging, edema, necrosis, thrombogenesis, hypotension, and pain are observed immediately after snakebites involving Viperidae or Crotalinae. These symptoms are caused by a bioactive mixture of proteinases, phospholipase A_2_, phosphodiesterase, 5′-nucleotidase, L-amino acid oxidase, peptides, and C-type lectins [1] Proteinases are considered to be one of the main elements that cause these symptoms. Proteinases are present in the venoms of many snakes and are structurally classified into trypsin-type snake venom serine proteases (SVSP) and snake venom metalloproteinases (SVMP).

SVMP are organized into three classes according to their size and domain composition [2]. Phylogenetically, SVMP are most closely related to the mammalian ADAM (a disintegrin and metalloproteinase) family [3]. Class P-I SVMP are the smallest SVMP, as they only have a metalloproteinase (M) domain. Class P-II SVMP contain a disintegrin-like (D) domain, which is connected by a short spacer region to the carboxyl terminus of the M domain [4]. Class P-III SVMP contain a cysteine-rich (C) domain on the carboxyl side of the D domain.

*Protobothrops* (habu) is classified into the Crotalinae family, and *P. flavoviridis* (Hon habu), *P. elegans* (Sakishima habu), and *P. tokarensis* (Tokara habu) are all found in Japan. Snake venom metalloproteinases (SVMP) account for at least 30% of the components of most *Protobothrops* venoms, suggesting that SVMP play significant roles in envenomation-related pathologies, such as bleeding, intravascular clotting, edema, thrombogenesis, inflammation, and necrosis [5,6,7,8]. In particular, SVMP are the primary factors responsible for hemorrhaging [9]. A non-hemorrhagic H_2_ proteinase (PF-H_2_) and four hemorrhagic proteinases (HR1A, HR1B, HR2a, and HR2b) were isolated from *P. flavoviridis* venom collected in Okinawa. SVMP have various physiological functions, e.g., they exhibit fibrinolytic/fibrinogenolytic (FL) and hemorrhagic activity, activate prothrombin, induce apoptosis in vascular endothelial cells, and affect platelet aggregation. The relationship between the structures and functions of metalloproteinase/disintegrin/cysteine-rich (MDC) domains has been reported previously, and specific functions of SVMP were suggested to be dependent on the structures of these domains [3,7,10,11]. Structural comparisons between SVMP revealed differences in the features of the substrate-binding region of the M domain; however, no relationship was found between these structural differences and hemorrhagic activity [12]. This article describes the structures and FL, hemorrhagic, and platelet aggregation-inhibiting activities of SVMP that were isolated from *Protobothrops* (*P. flavoviridis*, *P. elegans*, and *P. tokarensis*) venoms collected in Japan.

## 2. Classification of the Domain Structures of SVMP

**Figure 1:**
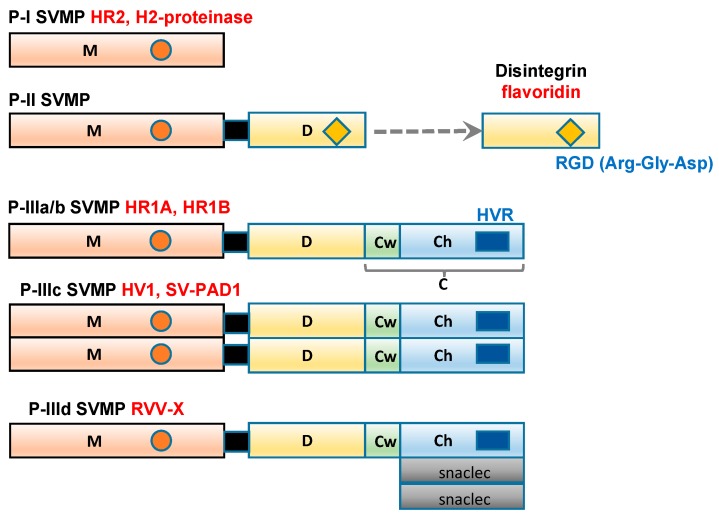
Schematic diagram of the domain structures of P-I, P-II, and P-III SVMP Each domain or subdomain is represented by a different color. M: metalloprotease domain (orange); D: disintegrin domain (yellow); C: cysteine-rich domain; Cw: the cysteine-rich “wrist” subdomain (light green); Ch: the cysteine-rich “hand” subdomain (light blue); HVR: hypervariable region (dark blue); snaclec: snake venom C-type lectin-like domain (grey).

SVMP range in size from 20 to 100 kDa and have been classified into three groups, P-I to P-III (Figure 1), according to their domain structures [13]. Class P-I SVMP are the smallest SVMP, as they only contain an M domain (position 1–214). Class P-II SVMP contain a canonical D domain (position 219–307), which is connected by a sort spacer region to the carboxyl terminus of the M domain. Class P-III SVMP contain a C domain (position 308–432), which is located on the carboxyl side of the D domain. Single-chain class P-III SVMP, including the HR1A and HR1B isolated from *P. flavoviridis* venom [14,15], have been subclassified as P-IIIa. In addition, P-III SVMP have been further divided into subclasses based on their post-translational modifications, such as whether they are subjected to dimerization (P-IIIc) or proteolytic processing (P-IIIb). P-IIId SVMP, for example, a specific factor X activator derived from Russell’s viper (*Daboia russelli*) venom (RVV-X) contains an additional C-type lectin-like domain [16,17].

## 3. Structures and Characterization of SVMP

**Figure 2:**
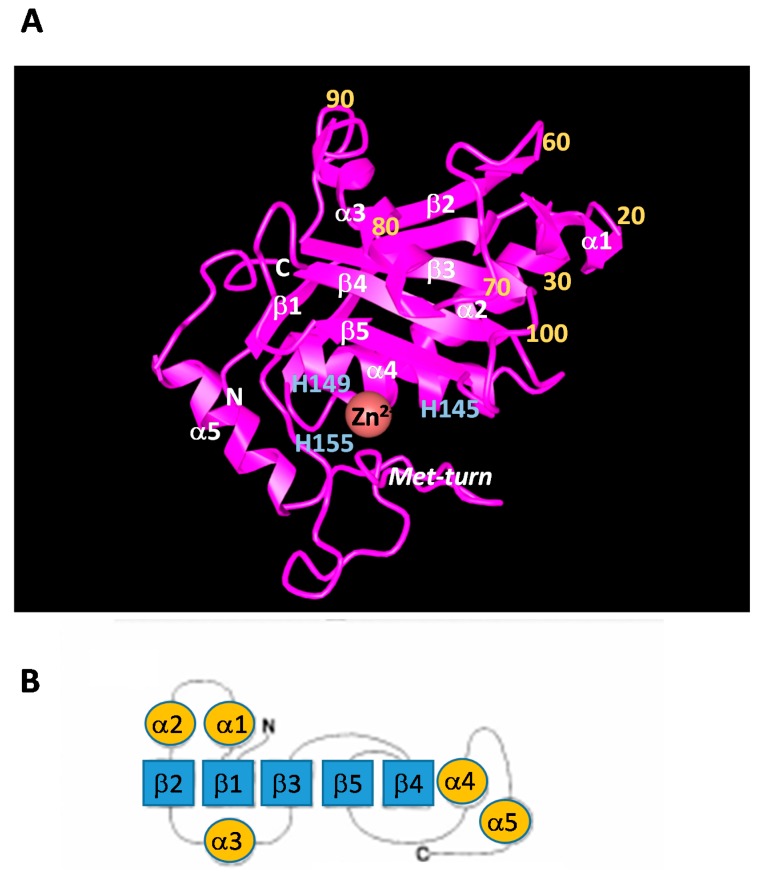
(**A**) Three-dimensional structure of the M domain of an H_2_-proteinase derived from *Protobothrops flavoviridis* venom [19]. α1–α5: α-helices; β1–β5: β-sheet; Yellow number shows amino acid position; Light blue shows His residues in Zn^2+^-binding sequence; (**B**) Topological packing diagrams for H_2_-proteinase derived from *P. flavoviridis* venom.

**Figure 3:**
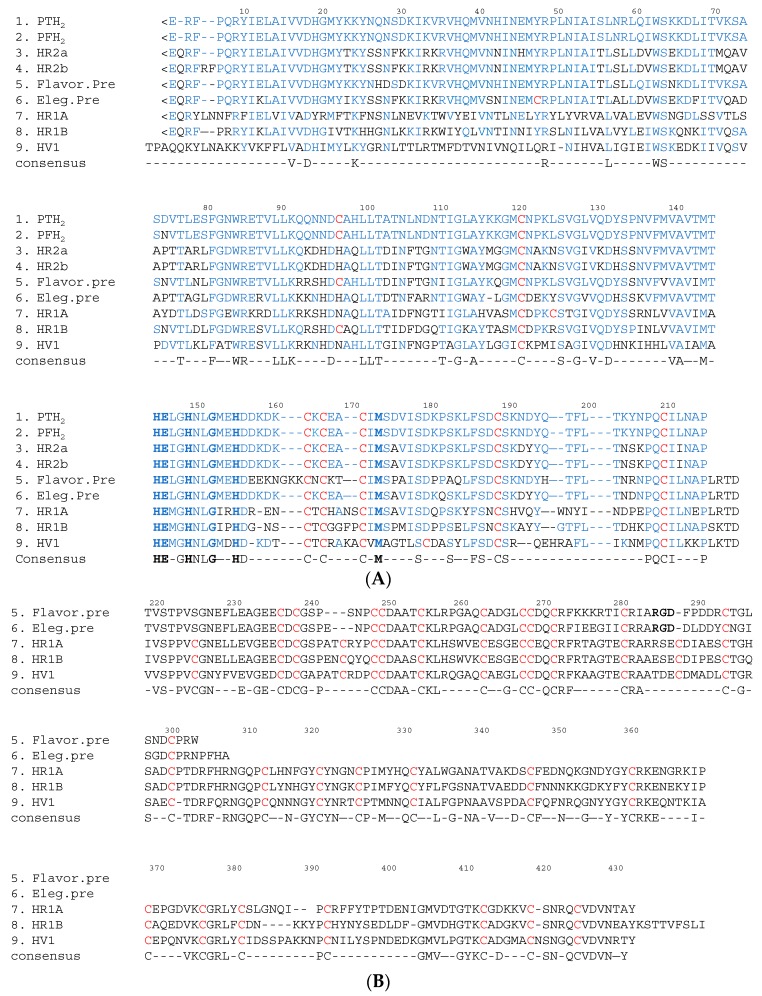
Comparison of the amino acid sequences of PT-H_2_ with those of other *Protobothrops* venom metalloproteinases. (**A**) Amino acid sequences of the M domains of SVMP; (**B**) Amino acid sequences of the DC domains of P-II and P-III SVMP. Gaps have been introduced to facilitate comparisons of sequence similarity. <E is pyroglutamic acid. The cysteine residues are shown in red. The binding site for Zn^2+^ and the Met-turn amino acid residues are shown in bold. The amino acid sequence of PT-H_2_ and the conserved amino acid residues are shown in blue. M domain: position 1–214; D domain: position 219–307; C domain: position 308–432; PTH_2_: a non-hemorrhagic P-I SVMP derived from *P. tokarensis* venom [20]; PFH_2_: a non-hemorrhagic P-I SVMP derived from *P. flavoviridis* venom [21]; HR2a [22] and HR2b [23]: hemorrhagic P-I SVMP derived from *P. flavoviridis* venom; Flavor. pre: a flavoridin precursor (a disintegrin) derived from *P. flavoviridis* venom [24]; Eleg. pre: a precursor of elegantin (a disintegrin) derived from *P. elegans* venom [25]; HR1A and HR1B [14]: hemorrhagic P-IIIa/b SVMP derived from *P. flavoviridis* venom; HV1 [26]: an apoptotic P-IIIc SVMP derived from *P. flavoviridis* venom

The M domain of SVMP contains a conserved Zn^2+^-binding sequence (His-Glu-X-X-His-X-X-Gly-X-X-His; positions 145–155) and a Met-turn (Met^173^) bearing the typical structural features of the metzincin family of metalloproteinases [18]. The amino terminus of the upper M domain of PF-H_2_ [19] has a central core consisting of a highly twisted five-stranded β-sheet and four β-helices (Figure 2). The carboxyl terminus of the lower M domain consists of a C-terminal helix preceded by an irregular region. This irregular region is presumably important for substrate recognition because it forms part of the wall of the substrate-binding pocket [3]. In SVMP, about 26%, 40% and 50% of the amino acid sequences of the M, D, and C domains, respectively, are conserved (Figure 3). As for the amino acid sequences of the SVMP found in *Protobothrops* venom, most differences were detected in the M domain. In particular, most of the variation was found in three regions (positions 26–35, 70–79 and 92–117) (Figure 3A). As shown in Figure 2, positions 26–35, 70–79 and 92–117 are located in α2-, α3-helices, and β3-sheet, respectively. It is suggested that these three regions influence the substrate specificity of P-I SVMP.

P-I SVMP are single-chain polypeptides with molecular masses of about 25 kDa (200–210 amino acid residues). The proteolytic activities of these enzymes are not influenced by *p*-APMSF (*p*-amidino-phenylmethylsulfonyl fluoride hydrochloride, an inhibitor of serine proteases), but are completely inhibited by EDTA (ethylenediaminetetraacetic acid). P-I SVMP are the smallest SVMP, as they only have an M domain. Therefore, the hemorrhagic and proteolytic activities of P-I SVMP are generally weaker than those of P-III SVMP, which have a C domain containing a hypervariable region (HVR) (positions 382–403 in Figure 3B). The HVR segment is considered to recognize substrates for P-III SVMP during protein-protein interactions [3,27,28].

P-II SVMP contain a canonical D domain, which is connected by a sort spacer region to the carboxyl terminus of the M domain. In general, snake venom disintegrins are generated by the proteolytic processing of large precursor P-II SVMP [29,30,31], although there is an exception to this rule [32]. Disintegrins typically possesses an Arg-Gly-Asp (RGD) recognition sequence in an extended loop (RGD loop), which inhibits integrin-mediated platelet aggregation and cell-matrix interactions [33,34].

P-III SVMP are classified into the following subclasses: P-IIIa/b, which exhibit hemorrhagic and proteolytic activity and inhibit platelet aggregation; P-IIIc, which induce apoptosis in human umbilical vein endothelial cells (HUVEC); and P-IIId, which activate factor X or prothrombin. When the amino acid sequences of the DC domains of class P-II integrin precursors (flavoridin [24] and elegantin [25]), class P-IIIa/b hemorrhagic SVMP (HR1A and HR1B [14]), and a P-IIIc HUVEC apoptosis inducer (HV1 [26]) were compared, it was found that the SVMP within the same class displayed a high degree of homology. The biological activities of SVMP are shown in Table 1. Interestingly, among the class P-III SVMP, the SVMP within the same subclass tended to have similar physiological functions. Furthermore, the HVR segments of these SVMP also demonstrated a high degree of homology among the P-III SVMP with similar physiological functions. It is suggested that the HVR segment might be an additional protein-binding site [3,27,28].

## 4. FL Activity

FL enzymes and thrombin-like enzymes are widely distributed among the venoms of Crotalinae and Viperidae. The FL enzymes and thrombin-like enzymes found in snake venom act on fibrinogen and fibrin, leading to the defibrinogenation of blood and fibrinolysis, which reduces blood viscosity [35,36,37]. The FL enzymes present in snake venom include both SVSP and SVMP. The FL activity of SVSP derived from *P. elegans* venom, such as elegaxobin I and II [38], preferentially results in the release of FPA from the fibrin α chain rather than FPB from the fibrin β chain, which causes incomplete fibrin clotting and a consequent increase in fibrinolytic activity. Furthermore, the fibrin fibers produced by thrombin-like SVSP are unstable and more susceptible to plasmin proteolysis than those produced by thrombin, and are therefore promptly degraded. TSV-PA belongs to a family of trypsin-type SVSP [39]. Tissue-type plasminogen activator-like SVSP, such as TSV-PA and the plasminogen activator present in *Lachesis muta* venom (LV-PA) [40], specifically cleave the Arg^561^-Val^562^ bond in plasminogen to generate two-chain plasmin, which results in fibrinolytic activity [41,42].

**Figure 4:**
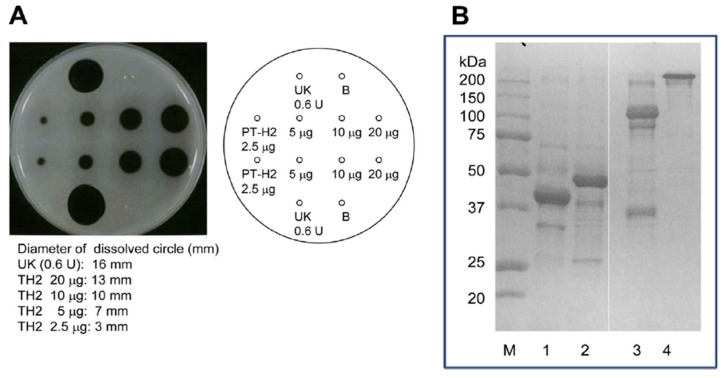
Effect of PT-H_2_ on bovine fibrin plates and SDS-PAGE of solutions that had been dissolved using PT-H_2_ or urokinase. (**A**) Fibrinolytic activity of PT-H_2_ and urokinase (as a positive control); (**B**) SDS-PAGE (Laemmli, 10% gel, Coomassie Brilliant Blue staining) of solutions that had been dissolved using PT-H_2_ (lane 2: reducing conditions, lane 4: non-reducing conditions) or urokinase (lane 1: reducing conditions, lane 3: non-reducing conditions). The assay of fibrinolytic activity was measured using the fibrin plate. A fibrin plate was prepared by the following methods. Bovine fibrinogen solution (16 mL, 10 mg/mL) in 0.02 M Tris-HCl buffer (pH 7.5) containing 0.15 M NaCl and 5 mM CaCl_2_ was poured in a plate (internal diameter: 94 mm). Then thrombin solution (100 μL, 100 units/mL) was added to the plate, and clotted at 30 °C for 2 h. Sample solution containing the protein applied to the well, and incubated at 25 °C overnight. After incubation, the fibrin plate was washed with saline three times, and then circle diameters dissolved by fibrinolytic enzyme and by urokinase as positive control were measured, respectively. Fluid from each circle dissolved by PT-H_2_ protease or urokinase was sampled and then subjected to SDS-PAGE.

The FL activity of SVMP involves the selective cleavage of the Aα chain of fibrinogen rather than the Bβ chain; however, these proteinases do not produce fibrin clots. On the other hand, this is not the case for some of SVMPs found in the venoms of *Trimeresurus mucrosquamatus* [43] or *Crotalus atrox* [44], which degrade the β chain and γ chain, respectively. Furthermore, P-I SVMP, such as a H_2_ proteinase derived from *P. tokarensis* venom (PT-H_2_) [20] and FE-32kDa (a 32-kDa fibrinolytic enzyme) from *Gloydius blomhoffii sinaiticus* venom [45], can promptly dissolve fibrin plates, although their fibrinolytic activity is weaker than that of urokinase (Figure 4A). Interestingly, the PF-H_2_ proteinase [21] found in *P. flavoviridis* venom, in which the Asp^75^ residue of the PT-H_2_ proteinase from *P. tokarensis* venom has been replaced with an Asn^75^ residue (Figure 3), is not able to dissolve fibrin plates [20]. While all of the PF-H_2_ proteinase molecules extracted from *P. flavoviridis* venom exhibited the same amino acid sequence, various sugar chains were found at position 75 (Asn-X-Thr). It is suggested that Asp^75^ is an important amino acid residue for fibrinolytic activation because PT-H_2_ proteinase has a different substrate specificity from PF-H_2_ proteinase.

The fibrinolytic activation mechanisms of urokinase and PT-H_2_ are obviously different. The results of sodium dodecyl sulfate polyacrylamide gel electrophoresis (SDS-PAGE) of fibrin that had been dissolved by PT-H_2_ or urokinase are shown in Figure 4B. Urokinase completely digested the α, β, and γ chains of fibrin. In addition, the fibrin polymer was completely digested by urokinase, and fraction Y (molecular mass: 150 kDa), which is a fibrinogen/fibrin degradation product produced by plasmin, was released. On the other hand, PT-H_2_ completely digested the α and β chains, but barely affected the γ chain or the fibrin polymer [20].

## 5. Hemorrhagic Activity

Marked hemorrhaging, edema, necrosis, thrombogenesis, hypotension, and pain occur immediately after snakebites involving Viperidae and Crotalinae. Hemorrhagic SVMP have been isolated from many snake venoms and are considered to be one of the causes of the abovementioned symptoms. In vivo, hemorrhagic SVMP act on the peripheral vasculature, which causes red blood cells to leak from the affected blood vessels. Therefore, the hemorrhagic SVMP present in the snake venoms of Viperidae and Crotalinae are considered to be lethal factors. HR2a and HR2b (P-I SVMP), which have low molecular weights, and HR1A and HR1B (P-III SVMP), which have high molecular weights, were isolated from *P. flavoviridis* venom and characterized in a previous study [46]. The H_2_ proteinase (P-I SVMP) from the same snake venom (PF-H_2_), which has a low molecular weight, does not exhibit hemorrhagic activity [47]. The hemorrhagic activities of HR2a and HR2b (minimum hemorrhagic dose (MHD): 66 ng in both cases) are about 6–10 times weaker than those of HR1A and HR1B (MHD: 11 ng and 6.7 ng, respectively). Interestingly, the amino acid sequences of the M domains of four SVMP [18,22,23] isolated from *P. flavoviridis* venom displayed a high degree of homology compared with those of SVMP from other snake venoms (Figure 3). The differences in the hemorrhagic activity of these SVMP suggested that the DC domains of HR1A and HR1B influence their hemorrhagic activity. The hemorrhagic and proteolytic activities of P-I SVMP are generally weaker than those of P-III SVMP, whose C domains contain an HVR segment (positions 382–403 in Figure 3B). On the other hand, PF-H_2_ and PT-H_2_ do not exhibit hemorrhagic activity, although the amino acid sequences of these P-I SVMP demonstrate a high degree of homology, and they also share about 77% homology with HR2a (Figure 3A). Furthermore, an array peculiar to amino acid sequences of non-hemorrhagic P-I SVMPs such as PF-H_2_ proteinase and PT-H_2_ proteinase were admitted. On comparison between two non-hemorrhagic P-I SVMPs and M domains of hemorrhagic SVMPs such as HR1A and 1B, HR2a and 2b, Lys^71^, Gln^93^, Asn^95^, His^99^, Leu^106^, and Lys^117^ of two non-hemorrhagic P-I SVMPs are replaced by the amino acid residues of Glu (or Thr), Lys (or Arg), His, Gln, Phe, and Gly (or Ala), respectively. Most of the replaced amino acid residues are amino acids that influence protein-protein interactions between basic or hydrophobic amino acids. It is suggested that the hemorrhagic effects of P-I SVMP are controlled by theese amino acid residues. These amino acid residues are an important target for functional analyses of P-I SVMP.

## 6. Inhibition of Platelet Aggregation

Platelet aggregation is a key event in thrombus formation and is dependent on the binding of adhesive proteins to αIIbβ3 integrin molecules on the platelet surface. Platelet adhesion proteins, such as fibrinogen and von Willebrand factor, contain the tripeptide segment Arg-Gly-Asp (RGD) as a cell recognition site. Snake venom proteins, including disintegrins and SVMP, inhibit platelet aggregation [48,49]. Snake venom disintegrins are a family of non-enzymatic proteins from the snake venoms of Crotalinae and Viperidae. They were shown to contain the RGD or Lys-Gly-Asp (KGD) sequence in a homologous position and to inhibit the interaction between fibrinogen and αIIbβ3 integrin. Snake venom disintegrins bind to αIIbβ3 integrin stronger than the RGD sequence of fibrinogen because the RGD sequences of these proteins are located on the top of the loop structure. Disintegrins were isolated from many snake venoms in the 1990s and have been actively researched [27]. In Japanese studies, four disintegrins, cytotoxic factor (CTF)-I, CTF-II [50], flavoridin [51], and triflavin [52], were detected in *P. flavoviridis* venom, and nine elegantin [25,53] isoforms were isolated from *P. elegans* venom.

On the other hand, P-I and P-III SVMP, which inhibit platelet aggregation, have been purified from the venoms of *P. flavoviridis* and *P. elegans.* In addition, HR1A, HR1B [22], and SV-PAD-2 [54], which are P-III SVMP, have been isolated from *Protobothrops* snake venom in Japan. HR1A and HR1B derived from *P. flavoviridis* venom undergo autoproteolysis and are released as single major fragments of 32 and 34 kDa, respectively, containing D and C domains. It is suggested that these fragments inhibit platelet aggregation [22]. The cDNA sequences of the D domain of flavoridin and the elegantin precursor [28,29] were classified as P-II SVMP [24] and exhibited a high degree of homology with the D domain of HR1A. Thus, it was suggested that snake venom disintegrins, such as flavoridin and elegantin, are generated from P-II or P-III SVMP after they have been cleaved autoproteolytically [24,25,26]. However, the RGD sequence of the disintegrin precursor is replaced with Arg-Ser-Glu, Glu-Ser-Glu, and Thr-Asp-Glu sequences in the D domains of HR1A, HR1B, and HV1, respectively (Figure 3B). Moreover, SV-PAD-2 from *P. elegans* venom had a marked effect on adenosine diphosphate (ADP)- and collagen-induced platelet aggregation (half maximal inhibitory concentrations [IC_50_]: 240 nM and 185 nM, respectively) and rapidly inhibited ADP-induced platelet aggregation. SV-PAD-2 can be subclassified as a P-IIIc SVMP based on the fact that it undergoes dimerization because its molecular mass according to SDS-PAGE was 110 kDa under non-reducing conditions and 52 kDa under reducing conditions [54].

Recently, it has been reported that P-I SVMP, such as barnettlysin-I (Bar-I) and *Bothrops moojeni* venom metalloproteinase (BmooMP) α-II, inhibit ADP-, collagen- and ristocetin-induced platelet aggregation. BmooMPα-II is a non-hemorrhagic P-I SVMP that inhibits platelet aggregation [55]. In addition, Bar-I is a 23,386 Da single-chain P-I SVMP that exhibits FL and hemorrhagic activity and inhibits platelet aggregation (IC_50_: 1.3 μM for ristocetin-induced platelet aggregation; 3.2 μM for collagen-induced platelet aggregation) [56]. Shanchez et al. suggested that Bar-I strongly inhibits platelet aggregation via the cleavage of α2β1 integrin, which is a selective collagen receptor [56]. Furthermore, we also recently found that PT-H_2_ inhibited ADP-induced platelet aggregation (IC_50_: 8 μM). As PT-H_2_ inhibits platelet aggregation to a different extent to Bar-I, it is likely that PT-H_2_ inhibits platelet aggregation via a different mechanism from Bar-I. Many of the platelet aggregation inhibitors isolated from SVMP are disintegrin domain-containing P-II or P-III SVMP. In a recent investigation, I found that PT-H_2_ inhibited ADP-induced platelet aggregation. I suggest that PT-H_2_ also inhibits platelet aggregation via the cleavage of platelet integrins.

## 7. Conclusions

In this paper, we have described the structures and functions of SVMP derived from *Protobothrops* species (*P. flavoviridis* (hon habu), *P. elegans* (sakishima habu), and *P. tokarensis* (tokara habu)) venom collected in Japan. The venoms of these snakes contain many SVMPs that exhibit a variety of physiological and biological functions. SVMPs range in size from 20 to 100 kDa and have been classified into three groups, P-I to P-III, according to their M, D, and C domain characteristics. P-III SVMP display various physiological functions associated with the DC domain. In the current study, it was suggested that the DC domain (especially the HVR segment in the C domain) influences the substrate specificity of SVMP, and the M domain is thought to function like a pair of scissors during substrate cleavage. However, P-I SVMP that displayed various substrate specificities were recently purified from several snake venoms.

As for the amino acid sequences of the SVMP found in *Protobothrops* venom, most differences were detected in the M domain, so it may be considered that the functions of SVMP are influenced by these particular amino acid residues in the M domain. The SVMPs isolated from all *Protobothrops* venoms collected in Japan exhibited similar structures. The SVMPs examined in the present study might be useful for clarifying the relationship between the M domain architecture and function.

## Figures and Tables

**Table 1:** Characterizations and biological activities of SVMPs from *Protobothrops* venom in Japan.

	Class	Domains	Sorce	Biological Activities	
HR1B	P-IIIa/b	MDC	*P. flavoviridis*	Hemorrhagic	
HR1A	P-IIIa/b	MDC	*P. flavoviridis*	Hemorrhagic	
HR-Ele-1	P-IIIa/b	MDC	*P. elegans*	Hemorrhagic	
HV1	P-IIIc	MDC (dimer)	*P. flavoviridis*	N.D.	Appoptotic
SV-PAD-2	P-IIIc	MDC (dimer)	*P. elegans*	Non-hemorrhagic	Inhibition of platelet aggregation
Flavoridin Pre.	P-II	MD	*P. flavoviridis*	N.D.	Precursor of disintegrin
Elegantin Pre.	P-II	MD	*P. elegans*	N.D.	Precursor of disintegrin
HR2a	P-I	M	*P. flavoviridis*	Hemorrhagic	
HR2b	P-I	M	*P. flavoviridis*	Hemorrhagic	
H_2_ proteinase	P-I	M	*P. flavoviridis*	Non-hemorrhagic	Proteolytic
PT-H_2_ proteinase	P-I	M	*P. tokarensis*	Non-hemorrhagic	Fibrinolytic, inhibition of platelet aggregation

N.D.: not determined.
